# Bcl-2 modifying factor (Bmf): “a mysterious stranger” in the Bcl-2 family proteins

**DOI:** 10.1038/s41418-025-01562-z

**Published:** 2025-08-23

**Authors:** N. V. Pervushin, D. K. Nilov, B. Zhivotovsky, G. S. Kopeina

**Affiliations:** 1 Engelhardt Institute of Molecular Biology, Russian Academy of Sciences, Moscow, Russia; 2 Faculty of Medicine, M.V. Lomonosov Moscow State University, Moscow, Russia; 3 Belozersky Institute of Physicochemical Biology, Lomonosov Moscow State University, Moscow, Russia; 4 Division of Toxicology, Institute of Environmental Medicine, Karolinska Institute, Stockholm, Sweden

**Keywords:** Cell biology, Cancer

## Abstract

Members of the Bcl-2 family are essential regulators of cell fate. Some of them (proapoptotic) promote cell death, while others (antiapoptotic) support cell survival. Bcl-2 modifying factor (Bmf) is an understudied BH3-only protein of this family that is widely expressed in many normal and cancer tissues. Bmf’s proapoptotic activity is essential in physiological and pathological processes, including hematopoiesis, gametogenesis, diabetes, tumorigenesis, etc. However, Bmf has remained in the shadow of other BH3-only proteins for many years. This review aims to rectify this injustice and elucidate the multifaceted functions of Bmf, its regulation, and its significance in both normal and pathological contexts.

## Facts

- The BH3-only protein Bmf is a key regulator of anoikis
- Bmf is involved in hematopoiesis and gametogenesis
- Dysregulation of Bmf contributes to the development of cancer and non-cancer diseases

## Open questions

- Are the functions of Bmf redundant in normal and malignant cells?
- Could Bmf be a prognostic biomarker for cancer progression?
- Which mechanisms underlie Bmf’s dysregulation in various processes?
- Could Bmf regulation be exploited in anticancer therapy?
- Is Bmf an “activator” or “sensitizer” of the BH3-only protein?

## Introduction

Apoptosis, the most investigated type of programmed cell death (PCD), is a complex process that is essential for the survival of multicellular organisms. Two main pathways of apoptosis induction are known: the extrinsic (receptor-mediated) pathway, which is induced by binding of death ligands (e.g., FasL) to corresponding death receptors (e.g., Fas), and the intrinsic (mitochondrial) pathway, controlled by the proteins of the Bcl-2 family [1, 2]. The members of this family possess anti- and proapoptotic properties. The prosurvival proteins (Bcl-2, Mcl-1, Bcl-xL, Bfl-1, Bcl-w, and Bcl-B) are responsible for neutralizing their proapoptotic counterparts, and they are commonly upregulated in tumor tissues and promote malignant progression. The proteins of the Bcl-2 family that trigger apoptosis are divided into two subsets: effectors and regulators. The first subgroup contains multidomain proteins (Bak and Bax) that can undergo conformational changes, oligomerize, form pores, and cause mitochondrial outer membrane permeabilization (MOMP). MOMP results in the activation of caspase signaling and subsequent apoptotic cell death. The second subgroup consists of single Bcl-2 Homology (BH) domain proteins and that is reflected in their name—BH3-only proteins. This subset is the most numerous of the Bcl-2 family proteins and comprises at least eight members (Bim, Noxa, Puma, Bad, Bid, Hrk, Bik, and Bmf) [3–9]. Some of them, like Bim, are well-studied and discussed in detail [10–13]. At the same time, the other BH3-only proteins, like Bmf, are poorly explored. Bmf was characterized in 2008 as a “minor brother” of Bim due to similarities in structure and function, but ever since, it has remained in the background [14]. Here, we attempt to remedy this omission and highlight the existing data concerning Bmf, which is now an underappreciated member of the Bcl-2 family proteins.

## General aspects of Bmf

Bmf protein, encoded by the *BMF* gene, which is located on chromosome 15q14/15q15.1, was identified as a novel BH3-only protein in 2001 using Mcl-1 as bait in a yeast two-hybrid (Y2H) screen [15]. The similarity between Bmf and Bim was quickly discovered [14]. Indeed, among other BH3-only proteins, these members of the Bcl-2 family have a lot in common: both proteins are constitutively expressed and active [14], in contrast to Bid, whose activation requires proteolytic cleavage [16] or Noxa and Puma, whose expression is mediated by p53 in response to stressful stimuli [17]. The unique feature of Bim and Bmf is the presence of motifs for binding dynein light chain proteins (DLC), which are involved in the transport of vesicles and organelles [15, 18, 19]. This circumstance determines similar localization of Bim and Bmf: under normal conditions, these proteins interact with DLC proteins (Bim – DLC1, Bmf – DLC2) and are sequestered to cytoskeletal structures (Bim – microtubules, Bmf – myosin V). In response to various stimuli, both proteins are released into the cytoplasm and, after translocation to mitochondria, endoplasmic reticulum (Bim), or only to mitochondria (Bmf), can regulate anoikis (an apoptosis-like form that will be described later) [14, 15, 20–22].

**Fig. 1:**
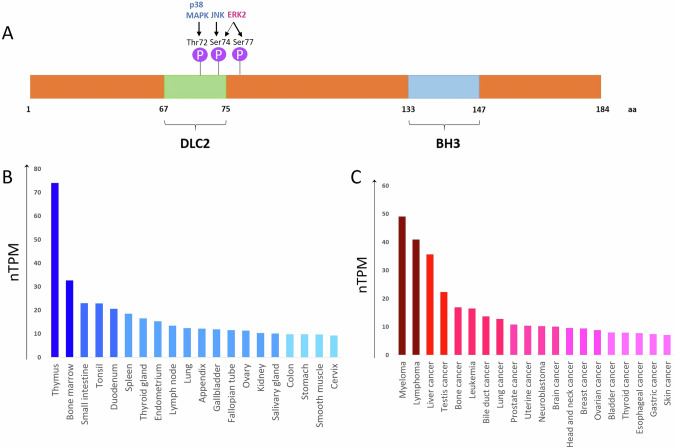
Bmf protein structure and its gene expression in human tissues. **A** The scheme of the Bmf domain structure was obtained from www.uniprot.org (Q96LC9). DLC2 - dynein light chain 2 motif; BH3 - Bcl-2 Homology (BH) domain 3. P – phosphosites of Bmf. Blue and orange colors of kinases indicate their impact on Bmf-mediated apoptotic activity discussed below – “pro-“ and “antiapoptotic” ones, respectively. The thin and thick arrows of ERK2 indicate “minor” and “major” phosphosites, respectively. **B** The consensus dataset of normalized *BMF* expression levels generated by mixing RNA-seq data from the HPA and GTEx projects in healthy human tissues (obtained from www.proteinatlas.org) [23, 166]. **C** The normalized *BMF* expression levels from the HPA cell line resource in human tumors (obtained from www.proteinatlas.org) [23, 166]. 20 healthy and cancer tissue types with the highest *BMF* expression level are visualized in the histograms. Data are presented as nTPM. HPA human protein atlas, GTE Genotype-Tissue Expression, nTPM normalized transcript per million.

Three variants of alternative splicing of human Bmf (Bmf I, Bmf II, and Bmf III) have been described. Bmf I, hereafter referred to as Bmf, is the most abundant isoform and contains 184 amino acids (aa) (Fig. 1A) [18]. *BMF* expression was detected in various normal and cancer cells [23]. According to the human protein atlas (HPA) database, Bmf mRNA is enriched in normal and pathological tissues and organs of the immune system (especially thymus, bone marrow, spleen), gastrointestinal tract, and reproductive system (Fig. 1B, C). For instance, the high expression of Bmf and its other splice variants (Bmf II and Bmf III) was predominantly observed in healthy and malignant B-cells [18]. This occurrence may be explained by Bmf’s impact on B-cell homeostasis, as discussed below. Both shortened isoforms that are predicted to encode proteins of 163 (Bmf II) and 129 (Bmf III) aa lack the BH3 domain but retain the DLC2 binding domain. Accordingly, they could not exert proapoptotic activity and were able to increase colony formation, thereby promoting cell survival, in contrast to Bmf [14, 18]. It is worth noting that, besides mammals, Bmf orthologs were also detected in fishes, frogs, rats, and mice, and their BH3 domain, as well as the DLC2 binding motif, are conserved in all these evolutionary distinct species [9, 24].

**Fig. 2:**
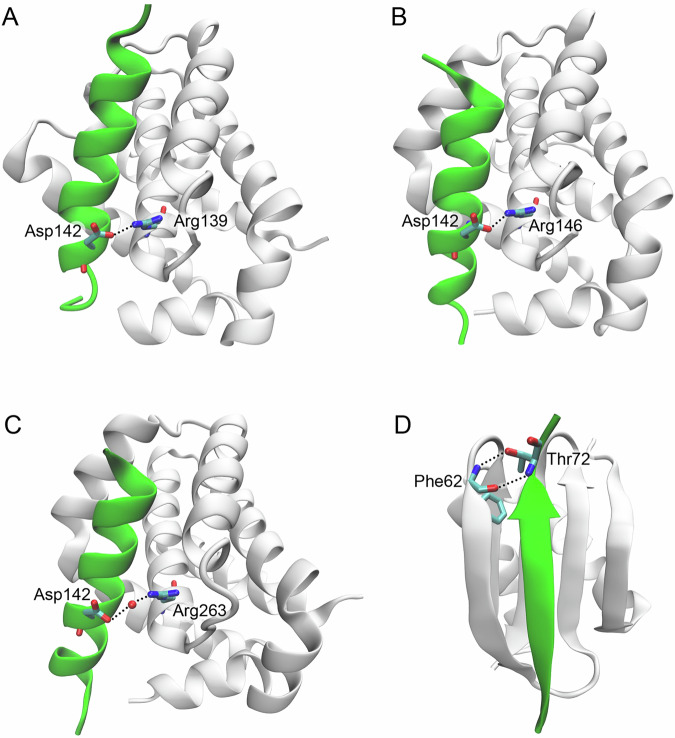
Crystal structures of Bmf with various proteins. Bmf in complexes with Bcl-xL (**A**), Bcl-2 (**B**), Mcl-1 (**C**), and DLC2 (**D**). Bmf fragments (green color) contain 127–147 aa (**A**–**C**) and 64–73 aa (**D**). The figure was prepared using VMD [167].

Despite its discovery more than 20 years ago, the exact protein structure of Bmf has not been determined, and only several complexes, containing part of its structure, were described in the literature and protein databases [25, 26]. Like other BH3-only proteins, Bmf interacts via its single BH domain with the special hydrophobic pocket, named the BH3-binding groove, of antiapoptotic members of the Bcl-2 family proteins [5]. Notably, Bmf was found to have a low affinity for Mcl-1 and Bfl-1/A1, which is similar to Mcl-1 [27], and predominantly interacts with Bcl-2, Bcl-xL, and Bcl-w [14, 20, 25, 28, 29]. The binding of α-helical fragments of human Bmf to the BH3-binding groove of Bcl-xL, Bcl-2, and Mcl-1 (PDB IDs 8iqk, 8iql, and 8iqm, respectively) was shown in Fig. 2A–C [25]. The interactions between these proteins are determined by the formation of conserved hydrophobic contacts (Ile133, Leu137, Ile140 and Phe144 residues of Bmf) and salt bridges (Asp142 residue of Bmf), which are formed in the Bmf-Bcl-xL (Asp142–Arg139) and Bmf-Bcl-2 (Asp142–Arg146) complexes (Fig. 2A, B) [25]. On the contrary, only a water-mediated hydrogen bond (Asp142-Arg263) instead of a salt bridge is formed in the Bmf-Mcl-1 complex (Fig. 2C) [25]. Accordingly, the Bmf aspartate residue is not found to directly bind to A1 protein in a mouse Bmf-A1 complex (PDB ID 2vog) [30]. Hence, the absence of electrostatic interactions may be a reason for lower Bmf affinity to Mcl-1 and A1 compared to Bcl-2, Bcl-xL, and Bcl-w [25, 30]. Additionally, the Bmf fragment corresponding to its DLC2 binding motif was found to adopt a β-strand conformation (Fig. 2D) and form multiple intermolecular hydrophobic and polar interactions. In particular, two hydrogen bonds between Thr72 residue of Bmf and Phe62 residue of DLC2 participate in stabilization of human Bmf-DLC2 complex (PDB ID 7cnu), and its destruction was associated with Bmf phosphorylation at Thr72 by mitogen-activated protein kinase (MAPK) p38, which is discussed below [26].

It should be noted that various pro- (e.g., Bim [13], Noxa [31]) and antiapoptotic (e.g., Bcl-xL [32], Mcl-1 [4]) members of the Bcl-2 family proteins are also regulated by alternative splicing. Importantly, this type of regulation could invert their functional activity in most cases: spliced isoforms of prosurvival proteins could acquire proapoptotic properties like Mcl-1S or Bcl-xS. By contrast, BH3-only proteins like Bmf II and III transcripts lose the BH3 domain, missing the ability to activate apoptosis. Unfortunately, these protein alterations are poorly studied, and the biological significance of many isoforms of the members of the Bcl-2 family, including Bmf, remains unclear. Nevertheless, alternative splicing seems to modulate the activity of Bcl-2 proteins, thereby determining cell fate.

It should be noted that alternative splicing is common for eukaryotic cells compared to non-AUG initiation translation [33]. Some time ago, a new alternative translation start site (CUG) was discovered in mouse Bmf mRNA, which resulted in two major isoforms: Bmf_S_ (an ortholog of the human protein, containing 185 aa) and Bmf_CUG_, containing 24 extra aa at its N-terminal end [34, 35]. Importantly, both mouse variants were proapoptotic and possessed similar properties, including subcellular localization and a pattern of interactions with Bcl-2 prosurvival proteins [34].

Notably, according to their functional activity, BH3-only proteins are commonly divided into two subgroups: “activators” (Bid-like), which can directly bind and activate effector proteins, and “direct sensitizers” (Bad-like), which can only disrupt complexes between pro- and antiapoptotic proteins to release Bax/Bak or “activators” [36]. However, this classification is somewhat controversial, as proteins vary in their ability to activate effectors, which is confirmed by the facts regarding Bmf. According to some reports, this protein was suggested to be a “sensitizer” [37, 38]. However, other studies indicate that Bmf might possess modest Bax/Bak-“activating capacity” [39–41]. Thus, Bmf could be considered a very mild “activator,” which is rather weaker than Bid or Bim, and its limited potency to activate Bax/Bak is likely to depend on the cell context or stimuli.

## Functions of Bmf in physiology and pathology

### Anoikis

Anoikis is an apoptotic-like type of cell death that is activated due to loss or inadequate cell adhesion [42]. This PCD subtype is of great importance in maintaining cell homeostasis in physiological and pathological conditions. As the first option, anoikis prevents carcinogenesis by eradicating cells that lose attachment to the extracellular matrix or adhere to inappropriate locations, thereby inhibiting the invasion of tumor cells. Anoikis suppression was found to be linked with cancer progression and metastasis in many reports [43–45]. At the same time, this process is also crucial for the development of normal tissues and organs. Particularly, anoikis-mediated regulation of cell rearrangement is associated with embryonic development, organ formation, tissue damage and repair, inflammatory response, and stem cell differentiation. The latter is especially important for further improvement of different stem cell‐based therapeutic applications, which are now intensively studied [46]. Therefore, besides cancer progression, anoikis dysregulation could be associated with various abnormalities that promote the development of cardiovascular diseases [47], diabetes [48, 49], brain injury [50, 51], tissue fibrosis [52, 53], and other non-cancer pathologies [46]. As mentioned above, apoptosis activation is under the tight control of multiple regulators, including members of the Bcl-2 family. The hallmarks of Bmf regulation at various levels will be discussed below in detail.

Importantly, starting with the first publication in 2001 [15], numerous studies reported that Bmf is one of the main mediators of anoikis, and as was mentioned above, Bmf’s liberation from the cytoskeleton leads to anoikis activation [21, 26, 54, 55]. Nevertheless, it should be noted that Bmf-lacking cells could also undergo anoikis, which illustrates the redundancy of Bcl-2 family proteins [56]. This phenomenon has great biological significance: a loss of one protein does not disrupt cell homeostasis, and its function could be substituted by others.

### Other PCD types

According to several papers, Bmf might be associated with the other PCD modes—autophagy and necroptosis. First, Bmf was also proposed to regulate autophagy via stabilization of Bcl-2/Beclin-1 interactions. In particular, Bmf suppression in a p53-dependent manner might facilitate the release of Beclin-1, one of the key autophagy mediators [57] from a complex with Bcl-2 and promote this PCD type [35, 58]. Furthermore, cells that lacked Bmf were associated with increased autophagy [35]. Thus, like other Bcl-2 family proteins, Bmf seems to be involved in the modulation of autophagic processes, acting as a “switch” between two PCD forms: upregulation of *BMF* expression or its release from microfilaments stimulates anoikis and suppresses autophagy.

Second, Bmf was assumed to participate in necroptosis. Initially, Bmf was suggested to be related to necroptosis regulation using genome-wide screening in L929 cells [59]. However, another report failed to confirm the participation of Bmf in necroptosis regulation. Tumor necrosis factor alpha (TNFα) and zVAD.fmk-mediated (a pan-caspase inhibitor) necroptosis was shown not to be altered in Bmf-deficient cells in comparison with parental L929 cells or mouse embryonic fibroblasts (MEFs) [60]. At the same time, Bmf knockout was shown to partially abate cadmium-induced necroptosis [60]. Thus, Bmf’s impact on necroptosis modulation is now controversial.

### Bmf in physiology

Analysis of Bmf knockout mice revealed that this protein, as well as the other BH3-only proteins, is dispensable for embryogenesis [56, 61]. However, Bmf suppression was shown to be engaged in the survival of hematopoietic stem and progenitor cells (HSPCs) [62]. It should be noted that Bmf plays an important role in B-cell development and maturation, and loss of Bmf causes abnormalities in B-cell homeostasis. In particular, it impaired apoptosis at various stages of B-cell development and resulted in the accumulation of pre- and mature B-cells [56, 61, 63]. Bmf knockout was also discovered to promote gamma-radiation-induced thymic lymphoma formation, which is in line with the HPA data (Fig. 1C, D) [56]. Moreover, double suppression of Bmf/Bim enhanced Bim-mediated alterations in B-cell homeostasis [64, 65] and could lead to premature lethality (~50% of mice were born alive) [65]. Thus, Bmf and Bim work in concert to regulate PCD and enhance each other’s actions.

Bmf was found to be involved in spermatogenesis and oogenesis regulation. On the one hand, Bmf expression was induced by a decline in testosterone levels in rat testis. Thus, cell death of spermatids following loss of cell attachment in response to lowered testosterone might be Bmf-dependent [24, 66]. On the other hand, this BH3-only protein could also regulate oogenesis: *BMF* depletion caused an increase in the number of primordial follicles from 100 postnatal days onward and extended fertility in mice [67]. Moreover, Bmf knockout led to enlarged germ cell numbers in embryonic and early postnatal mouse ovaries [68]. Interestingly, in contrast with males, which did not show any obvious developmental defects, female Bmf knockout mice are characterized by abnormalities of uterovaginal development [21, 64], including hydrometrocolpos and an imperforate vagina in about every fifth case [64]. The causes of this circumstance are probably associated with defects in apoptosis, which is essential to developing the female reproductive tract [69, 70]. In addition, Bmf is also implicated in mammary [55, 71] and intestinal epithelium morphogenesis [21]. For instance, elevated *BMF* expression was shown during anoikis in mammary [55] and intestinal epithelial cells (IEC), and Bmf knockout was found to rescue IECs in in vitro and in vivo models [21].

### Bmf in pathology

Bmf might also participate in the regulation of neurological disorders. First, its expression was disclosed to be significantly increased during oligodendroglia differentiation [72]. Second, Bmf could contribute to neuronal injury: Bmf deficiency resulted in neuroprotection of cortical neurons in response to excitotoxic or ischemic conditions [73] and NGF deprivation or amyloid beta toxicity [74]. Finally, Bmf-lacking mice were more susceptible to seizure-induced neuronal death compared to wild-type controls. As seizure activity could cause energy depletion, and apoptosis is an energy-dependent process, it might be speculated that this effect was not associated with apoptotic functions of Bmf, and could be explained, for instance, by necrotic death activation [75].

Bmf is likely to be involved in the pathogenesis of diabetes, particularly mediating apoptosis of renal proximal tubular cells [76, 77]. Interestingly, AMPK, a sensor of “bioenergetic stress”, was demonstrated to induce the expression of Bmf in pancreatic beta cells, as well as in neurons [75, 78, 79]. Moreover, Bmf could be activated in response to glucocorticoids and engaged in glucocorticoid-induced apoptosis [80]. At the same time, *BMF* depletion was observed to protect beta cells from apoptosis and enhance hyperglycemia in diabetic mice [79]. The exact mechanism of this phenomenon remains unclear. However, it should be noted that the overexpression of antiapoptotic proteins (e.g., Bcl-2 or Bcl-xL) led to similar effects (survival of beta-cells and augmented hyperglycemia) that might be explained by impaired glucose signaling [79, 81, 82]. Additionally, the Bmf level was disclosed to positively correlate with vascular calcification and aging in diabetic models, and *BMF* ablation was found to abrogate this effect, diminishing calcification and senescence of vascular smooth muscle cells [83, 84]. Generally, these findings suggest an important and understudied role for Bmf in the maintenance of glucose homeostasis.

As can be seen from the above, Bmf is implicated in the regulation of multifarious physiological (hematopoiesis, gametogenesis, tissue remodeling) and pathological (neuronal damage, diabetes) processes that are predominantly associated with apoptosis/anoikis modulation. Moreover, all findings presented here demonstrate non-redundant functions of Bmf compared to Bim and other BH3-only proteins, confirming its biological significance.

## The regulation of Bmf in normal and pathological conditions

### The transcriptional regulation of Bmf

**Fig. 3:**
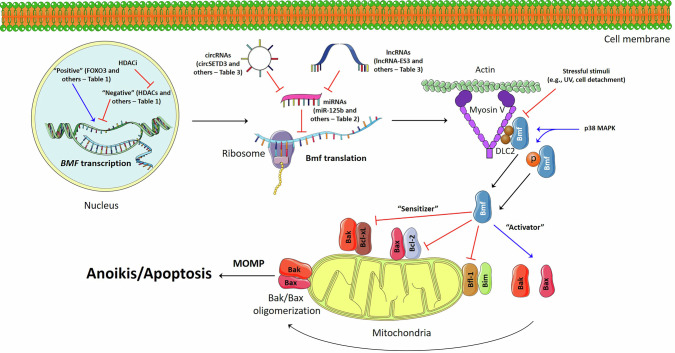
The regulation of Bmf protein structure and its participation in anoikis/apoptosis. P - phosphorylated form of the Bmf protein. The details and the other regulators, which are not presented here to simplify the scheme, are discussed in the text. The blue and red arrows indicate “positive” and “negative” influence on the participants of Bmf regulation and anoikis/apoptosis. This figure was generated by using the Servier Medical Art images, that is licensed under a Creative Commons Attribution 4.0 Unported License.

Various positive (e.g., FOXO3) and negative (e.g., YAP/TEAD/SLUG) transcription regulators, summarized in Table 1, have been proposed to modulate *BMF* expression (Fig. 3). Notably, besides that, a balance between histone acetylation and deacetylation, which is a part of epigenetic regulation, determines the expression levels of many genes in normal and cancer cells, and hyperacetylation of histones mediated by histone deacetylase inhibitors (HDACi) could induce transcription of various genes. Interestingly, numerous articles reported that several HDACi, including vorinostat (a drug approved by the FDA for the treatment of cutaneous T-cell lymphoma), were discovered to enhance *BMF* expression [85–92]. Specifically, HDACi-mediated apoptosis was associated with an elevation in the Bmf protein level, and Bmf knockout attenuated this effect. Moreover, HDAC1 [85] or HDAC8 [87] overexpression led to a decline in Bmf expression. Thus, HDACs serve as important transcriptional regulators of Bmf, and their inhibition could enhance cancer cell death through, among other matters, Bmf upregulation.

**Table 1:** The transcriptional regulation of Bmf.

Transcription factor	Influence on *BMF* gene expression	Biological effects and models
Smad4	Positive	Smad4-dependent Bmf induction during TGFβ-mediated apoptosis in hepatocyte cells [126]
FOXO3	Positive	FOXO3 inhibition led to decreased Bmf expression and promoted tumor growth and metastasis in E-cadherin-negative breast cancer cells [127]
STAT3	Positive	JAK1-mediated STAT3 activation caused enhanced Bmf expression in mammary epithelial cells [71]
	Negative	HDAC8, in cooperation with STAT3, was found to suppress *BMF* expression in colon cancer cells [87]
Eomesodermin (Eomes)	Negative	Eomes overexpression resulted in the downregulation of Bmf and augmented cell viability, while Eomes suppression had the opposite effect on colorectal cancer cells [128]
Heterogeneous Nuclear Ribonucleoprotein F (HNRPF) (RNA binding protein)	Negative	HNRPF, stimulated by insulin, bound HNRPF-responsive elements in the *BMF* promoter, blocking its transcription, and preventing Bmf-mediated apoptosis of renal proximal tubular cells (RPTC) in the diabetic kidney [77]
YAP signaling pathway	Negative	YAP/TEAD/SLUG complex inhibited Bmf transcription and abrogated cell death in EGFR-mutant non-small cell lung cancer (NCSLC) cells [118]
		YAP/TAZ suppressed KRAS inhibitor-mediated apoptosis via downregulation of Bmf and other BH3-only proteins in several tumor models [119]
IRF4	Negative	IRF4 suppressed Bmf and promoted cell survival of multiple myeloma cells [129]
c-Myb	Negative	c-Myb blocked Bmf expression, regulating cell survival during B lymphopoiesis [130]
ATF5	Negative	ATF5 induced anoikis resistance and promoted metastasis via Bmf suppression in neuroblastoma cells [131]
FOXM1	Negative	FOXM1 bound to the intronic cis-regulatory element of *BMF* and repressed it, causing anoikis evasion, while FOXM1 inhibition led to Bmf-mediated enhancement of cell death upon antimitotic drugs in human dermal fibroblasts [132]
HDAC4/RelB/p52	Negative	The RelB/p52 complex could interact with the *BMF* promoter and suppress transcription of its gene in the presence of HDAC4, which acetylates histones in this locus, but this mechanism is abolished in the absence of HDAC4, causing an increase in Bmf expression in multiple myeloma [92]

### The post-transcriptional regulation of Bmf

In contrast to the abovementioned alternative splicing of Bmf, which remains understudied, the regulation of mRNA levels of this BH3-only protein has become clearer during recent years. Above all, plenty of microRNAs (miRNAs) were shown to target Bmf (Fig. 2). These regulatory elements represent small (about 21–23 nucleotides) non-coding RNAs, which bind to corresponding mRNAs through the 3′-untranslated region (UTR), thereby causing their degradation [93]. Table 2 includes information about several miRNAs that participate in the negative control of *BMF* expression.

**Table 2:** Negative regulation of Bmf expression by various miRNAs.

miRNAs	Models
miR-125b	Glioblastoma [133], hematopoietic stem cells (HSCs) [134], esophageal squamous cell carcinoma (ESCC) [135], fibroblasts [136], macrophages [137]
miR-181c-5p	Cortical neurons [138]
miR-183-5p	Bladder cancer [139]
miR-185-5p	Ovarian cancer [140]
miR-19a	Mouse germ cells [141]
miR-193a-5p	Hepatocellular carcinoma (HCC) [142]
miR-197	Non-small cell lung cancer (NSCLC) [143]
miR-221	HCC [144, 145], ovarian cancer [146]
miR-222	Glioblastoma [147]
miR-29a	Rat uterus [148], lens epithelial cells [149]
miR-29b	Mouse neurons [150], osteoclasts [151]
miR-29b-3p	Rat myocardial cells [152], mice neurons [153]
miR-3156-3p	Leukemia [154]
miR-34c-5p	NSCLC [155], human aorta vascular smooth muscle cells (HA‐VSMCs) [84]
miR-421	Cholangiocarcinoma [156]
miR-450-5p	Goat granulosa cells [157]
miR-640	Glioblastoma [158]
miR-671-5p	Neuroblastoma [159]
miR-874-3p	Neuroblastoma [160]

Besides miRNAs, other post-transcriptional regulators were reported to orchestrate Bmf mRNA stability, which is responsible for the modulation of miRNA activity. These modulators mainly include long non-coding RNAs (lncRNAs) and circular RNAs (circRNAs). LncRNAs comprise molecules containing more than 200 nucleotides without protein-coding ability [94]. Moreover, there is a subgroup of antisense lncRNAs, which are transcribed in an antisense orientation compared to corresponding protein-coding or non-coding genes [95]. CircRNAs are single-stranded molecules, in which the 3′ and 5′ ends, unlike those in linear RNA, are covalently closed [96]. LncRNAs, circRNAs, and other regulators that contribute to the control of Bmf mRNA levels are presented in Table 3.

**Table 3:** Positive post-transcriptional regulators of Bmf expression.

Regulators	Mechanism of action	Biological effects and models
lncRNA SNHG14	Sponging of miR-181c-5p	SNHG14 promoted neuron injury in the ischemic stroke model [138]
lncRNA Gas5	Inhibition of miR-222	Gas5 exerted tumor suppressive functions in glioblastoma [147]
lncRNA‐ES3	Sponging of miR-34c-5p	lncRNA‐ES3 promoted calcification and senescence of HA‐VSMCs [84]
lncRNA RP11-521C20.3/BMF-AS1	Protection of the BMF mRNA	Inhibition of BMF-AS1 and BMF diminished calcification and senescence of vascular smooth muscle cells in diabetic mice [83]
CircSETD3	Sponging of miR-421	CircSETD3 blocked proliferation and induced apoptosis in cholangiocarcinoma cells [156]
CircSLC39A8	Sponging of miR-185-5p	CircSLC39A8 inhibition led to paclitaxel resistance in ovarian cancer [140]
Circ_0001360	Sponging of miR-671-5p	Circ_0001360 downregulation reduced neuroblastoma cell injury in the ischemic stroke model [159]
RNA-binding protein RBMS2	Enhancement of Bmf mRNA expression and stability via direct binding to the ARE sites in the 3′-UTR of its mRNA	RBMS2 caused Bmf-mediated sensibilization of breast cancer cells to doxorubicin [161]
Rho GTPase-activating protein (GAP) STARD13 3′UTR	Downregulation of miR-125b	STARD13 3′UTR led to Bmf-mediated apoptosis induction in breast cancer cells [162]
Sp110 nuclear body protein	Downregulation of miR-125b	Sp110 was shown to elevate Bmf levels and activate macrophage apoptosis, mediating macrophage resistance to *Mycobacterium tuberculosis* [137]

In addition, the RelB/p52 complex mentioned earlier was found to downregulate the Bmf level not only at the transcriptional level but also at the post-transcriptional stage. Specifically, this complex was able to interact with the promoter region of the miR-221 gene and augment its expression, while this miRNA was shown to target Bmf mRNA in different models, including multiple myeloma [92].

### The posttranslational regulation of Bmf

Above all, the level of Bmf can be altered in response to various stimuli. First, Bmf induction was observed upon cytokine withdrawal in granulocytes [97]. Later, Bmf suppression was found to reduce apoptotic cell death in response to impairment of CAP-dependent translation, in particular, under serum deprivation [34]. Besides that, Bmf was shown to mediate anoikis/apoptosis upon UV irradiation [15], paclitaxel (microtubule inhibitor) [98], and arsenic trioxide [99], which can also disturb microtubule dynamics [100]. Specifically, UV exposure resulted in a release of Bmf from cytoskeleton [15], and paclitaxel treatment led to a disruption of Bim complexes with prosurvival Bcl-2 proteins by a cooperation of Bmf and Puma in breast cancer [98]. At the same time, arsenic trioxide led to an increase in *BMF* expression in multiple myeloma [99]. Next, chemokines (CXCL12 and CCL21) and their corresponding receptors (CXCR4 and CCR7) were disclosed to inhibit anoikis and promote metastasis and tumorigenesis via the downregulation of Bmf levels. The precise mechanism of this phenomenon remains unknown and might be linked to the activation of several signaling pathways [101].

Furthermore, MAPK signaling cascades could contribute to the posttranslational regulation of Bmf. On the one hand, p38 MAPK, involved in a “proapoptotic” pathway [102], was found to affect Bmf and increase its apoptotic activity via p38 MAPK-mediated Bmf phosphorylation at Thr72, which is located within the DLC2 binding domain. This modification led to the disruption of interactions between DLC2 and Bmf, causing, as mentioned above, the release of the latter from the cytoskeleton and promoting cell death (Fig. 3) [26]. Another “proapoptotic” pathway, JNK [103], was observed to phosphorylate Bmf at Ser74; however, JNK was only found to facilitate Bmf-induced anoikis, but generally, it is not essential for anoikis induction [64, 104, 105]. On the other hand, ERK1/2, which positively regulates a “prosurvival” pathway [106], was disclosed to diminish the ability of Bmf to trigger apoptosis at different regulatory levels. First, suppression of ERK1/2 positive regulators NRAS or MEK was shown to elevate both Bmf mRNA and protein levels [107]. Second, activation of PI3K/AKT or MEK/ERK signaling pathways was associated with abatement of *BMF* transcription [55, 108]. Third, ERK2 was able to directly phosphorylate this BH3-only protein at Ser74 (“minor site”) and Ser77 (“major site”), which did not affect Bmf stability and its interaction with DLC2 or antiapoptotic Bcl-2 proteins. However, ERK2-mediated Bmf phosphorylation decreased its apoptotic activity [109]. Importantly, Ser74 was the same target site for JNK, but, obviously, the two-site phosphorylation demonstrated in this case could lead to the opposite results. Nevertheless, it might explain why Bmf was able to translocate from the cytoskeleton into the cytoplasm only in MEK inhibitor-sensitive cancer cells, while in resistant cells, this protein remained bonded to DLC2 and did not induce apoptotic cell death [110]. Finally, the EGFR/MEK/ERK pathway was also revealed to be activated and block anoikis via suppressing Bmf expression under hypoxic conditions [111]. The possible mechanism underlying this result remains to be elucidated, but the obtained findings suggest that tumors, which often survive under hypoxia due to the “Warburg effect,” can escape anoikis, proliferate, and be intolerant to Bmf-induced chemotherapeutics.

As mentioned earlier, DLC1/2 proteins can interact with Bmf and Bim, controlling their functional activity [15, 19, 38]. Recently, similarly to Bim and DLC1 [112], Bmf was found to dimerize and form a complex upon DLC2 binding, which might also be necessary for preventing its proteasomal degradation [38]. Moreover, these proteins were detected to assemble multiple complexes like Bmf-DLC-Bim, and Bmf overexpression was revealed to trigger Bim degradation [38]. Hence, these data suggest that “relative proteins” Bmf and Bim could modulate each other’s functions in the presence of DLC1/2 proteins.

## The role of Bmf in tumorigenesis

According to the findings observed, Bmf, like all proteins participating in the triggering of PCD, is under strict control at different levels. This complex regulatory system is essential to prevent spontaneous cell death. However, the alterations in Bmf regulation could promote the progression of both cancer and non-cancer diseases. On the one hand, an increase in the Bmf level due to the predominance of its “activators” might lead to excessive apoptosis in some pathological conditions, e.g., ischemic stroke. On the other hand, Bmf suppression (e.g., due to increased levels of miRNAs or decreased levels of “positive” regulators) could facilitate tumor growth and carcinogenesis, confirming the oncosuppressive role of Bmf that was illustrated in this review.

Next, in agreement with the previously mentioned Bmf’s impact on B-cell homeostasis, this BH3-only protein was observed to participate in the pathogenesis of chronic lymphocytic leukemia (CLL), which is associated with B-cell malignancy. First, a low level of Bmf was found in neonatal B1 B-cells in contrast with B2 B-cells, and Bmf deficiency coupled with high expression of c-Myc in B1 B-cells might predispose to CLL development [113]. Second, Single Nucleotide Polymorphism (SNP) rs539846-A, located in the third intron of *BMF*, was shown to diminish *BMF* expression via alteration of the RELA-binding motif and its enhancer activity. Hence, the presence of this genetic variant could also intensify oncogenicity in CLL [114].

Notably, a loss of 15q14/15, containing the *BMF* gene, was found in several metastatic tumors (lung, breast, and colorectal carcinomas) [115]. Considering that Bmf is involved in the regulation of mammary morphogenesis [55], this protein may be a possible prognostic marker in breast cancer. Logically, cancer cells evade Bmf-mediated anoikis, an important defense mechanism, and survive after detachment from their niches to progress and metastasize.

Interestingly, novel studies have shown a link between Bmf and tumorigenicity in liver cancer [116, 117]. As mentioned above, the YAP signaling pathway acts as a negative regulator of Bmf at the transcriptional level [118, 119]. Recently, YAP activation or Bmf inhibition was found to promote the stemness of liver cancer stem cells. Moreover, in conditions that mimic raised compression in growing tumors, the YAP/Bmf axis mediated enhanced tumorigenic potential, while Bmf overexpression was discovered to diminish these effects [116, 117]. Altogether, these results support the oncosuppressive functions of Bmf. Indeed, the loss of Bmf results in the protection of cancer cells from anoikis, facilitating metastatic spreading and tumorigenesis, but its overexpression or augmented release from cytoskeletal structures could trigger cell death and prevent metastasis of detached cancer cells.

It should be noted that malignant cells can accumulate mutations during tumorigenesis. Interestingly, mutant p53 (p53-R273H), which loses its oncosuppressive activity and acquires oncogenic properties [120], was shown to inhibit Bmf expression through activation of the PI3K/AKT signaling cascade in various cancer types [121], which corresponds with the previously mentioned report concerning the negative influence of this pathway on Bmf function [55]. Hence, p53 status may be a potential biomarker that predicts the proapoptotic activity of Bmf.

Finally, Bcl-2 family proteins can also undergo mutation, which alters their properties [5, 122]. However, Bmf mutations are not common in tumors [123, 124]. According to an open database (www.cbioportal.org) containing the results of multiple cancer studies, 122 genetic alterations of the *BMF* gene, predominantly deep deletions and mutations, were detected in 23 cancer types among a total of 32 (10,953 patients). The alterations were found most frequently in colorectal, ovarian, and lung carcinomas in absolute number (*n* = 14) and uterine carcinosarcoma in relative number (8.77%), which agrees with the disturbance of Bmf mRNA in cancer tissues, presented earlier (Fig. 1D). The detailed results are presented in Table 4.

**Table 4:** The frequency of BMF genetic alterations (www.cbioportal.org).

Cancer type	Number of cases	Alteration frequency			
		Deep deletion (78 cases)	Mutation^a^ (33 cases)	Amplification (11 cases)	Total (*n* = 122)
Colorectal adenocarcinoma	594	8 (1.35%)	6 (1.01%)	-	14 (2.36%)
Ovarian serous cystadenocarcinoma	584	13 (2.23%)	-	1 (0.17%)	14 (2.4%)
Lung adenocarcinoma	566	13 (2.3%)	1 (0.18%)	-	14 (2.47%)
Breast invasive carcinoma	1084	9 (0.83%)	1 (0.09%)	3 (0.28%)	13 (1.2%)
Uterine corpus endometrial carcinoma	529	4 (0.76%)	7 (1.32%)	1 (0.19%)	12 (2.27%)
Skin cutaneous melanoma	444	5 (1.13%)	5 (1.13%)	-	10 (2.25%)
Bladder urothelial carcinoma	411	2 (0.49%)	3 (0.73%)	1 (0.24%)	6 (1.46%)
Prostate adenocarcinoma	494	4 (0.81%)	-	1 (0.2%)	5 (1.01%)
Uterine carcinosarcoma	57	3 (5.26%)	1 (1.75%)	1 (1.75%)	5 (8.77%)
Mesothelioma	87	4 (4.6%)	-	-	4 (4.6%)
Stomach adenocarcinoma	440	1 (0.23%)	3 (0.68%)	-	4 (0.91%)
Glioblastoma multiforme	592	2 (0.34%)	1 (0.17%)	1 (0.17%)	4 (0.68%)
Sarcoma	255	1 (0.39%)	-	1 (0.39%)	2 (0.78%)
Kidney renal papillary cell carcinoma	283	2 (0.71%)	-	-	2 (0.71%)
Diffuse large B-cell lymphoma	48	2 (4.17%)	-	-	2 (4.17%)
Kidney renal clear cell carcinoma	511	1 (0.2%)	1 (0.2%)	-	2 (0.39%)
Cervical squamous cell carcinoma	297	1 (0.34%)	1 (0.34%)	-	2 (0.67%)
Liver hepatocellular carcinoma	372	1 (0.27%)	1 (0.27%)	-	2 (0.54%)
Brain lower grade glioma	514	-	1 (0.19%)	-	1 (0.19%)
Adrenocortical carcinoma	91	1 (1.1%)	-	-	1 (1.1%)
Esophageal adenocarcinoma	182	-	-	1 (0.55%)	1 (0.55%)
Acute myeloid leukemia	200	-	1 (0.5%)	-	1 (0.5%)
Lung squamous cell carcinoma	487	1 (0.21%)	-	-	1 (0.21%)

^a^34 mutations were observed in 33 cases (one case possessed 2 mutations): missense – 22, nonsense – 6 (*), frameshift deletion – 3, frameshift insertion – 1, splice site – 2. Arg135Gln/* was the most frequent amino acid alteration (3 times); Ser4Tyr, Gln48His/*, Pro105His/Leu, and Arg158Cys/His were detected twice. Among all mutations, only 3 alterations mentioned below were located in the functional regions of Bmf: DLC2 motif or BH3 domain.

All genetic variants can be considered variants of uncertain significance. Selected studies contain structural variants, mutations, and copy number data (TCGA PanCancer Atlas Studies from www.cbioportal.org) [163–165].

Nevertheless, despite their rarity, the appearance of genetic alterations in the Bmf gene may result in anoikis resistance and tumor progression. Thus, mutations in the DLC2 motif (Leu73Ile) or the BH3 domain (Arg135Gln/*; Gln138*) might lead to a loss of its ability to interact with other proteins, including members of the Bcl-2 family. For example, the mutation of Arg135 could change the spatial position of Ile133 and Leu137 in the BH3 domain of Bmf, which are important for the hydrophobic contacts with the BH3-binding groove of antiapoptotic Bcl-2 family proteins. Additionally, Leu73 directly binds with Thr72 in the DLC2 motif, which promotes stabilization of the human Bmf-DLC2 complex (Fig. 2D); consequently, the substitution Leu73Ile could destroy this complex. Taken together, Bmf mutations could serve as possible prognostic markers in patients with various malignant diseases.

## Concluding remarks

Here, we summarize various aspects of Bmf functions and regulation in normal and pathological conditions. This BH3-only protein of the Bcl-2 family plays a crucial role not only in “classical” apoptosis but also in anoikis regulation, contributing to normal development and cancer prevention. Despite numerous reports concerning Bmf, its significance in various states remains undefined, which is associated with an abundance of BH3-only proteins. Thus, mice lacking Bmf could survive, indicating that BH3-only (mainly Bim) members of the Bcl-2 family could be replaced by compensate the functions of each other. Hence, it is difficult to assess the exact impact of Bmf on specific processes. Nevertheless, the presence of several abnormalities in Bmf knockout models mentioned earlier [21, 61–65] illustrates its importance for normal development. Therefore, these observations suggest that there was no complete substitute for Bmf. This assumption may also be supported by the multiplex regulation of Bmf. It would be a bit strange that an “auxiliary” protein is under the strict control of most modulators. Moreover, some of the Bmf regulators presented here, for instance, the YAP/TEAD/SLUG complex, were observed to have an exclusive influence on Bmf, but not on its “relative” Bim [118].

Next, loss of Bmf was found to promote tumorigenesis and drug resistance, suggesting that this protein may be a prognostic marker of some cancer diseases. As mentioned, Bmf is involved in HDACi-mediated apoptosis, and its rate could predict the response of cancer cells to them. Moreover, considering the negative impact of the MEK/ERK signaling pathway on Bmf, the use of MEK inhibitors could enhance the efficacy of cancer therapy in some cases; meanwhile, dysregulation of Bmf might be linked with the development of resistance to MEK inhibitors. Finally, the investigations of all BH3-only proteins and their interactions with prosurvival Bcl-2 proteins at appropriate times allowed the development of a novel class of drugs – BH3-mimetics, including Venetoclax (a selective Bcl-2 inhibitor), approved by the FDA [5].

Notably, targeting members of the Bcl-2 family is an attractive therapeutic approach that has been intensively studied over the last decades. Despite achieving some success in this field (Venetoclax approval), several problems remain unresolved. In addition to low therapeutic efficacy in most cases due to the abundance and “compensatory effect” of Bcl-2 family proteins, its blocking could lead to undesirable side effects. Specifically, thrombocytopenia and cardiotoxicity are associated with Bcl-xL and Mcl-1 inhibition, respectively [5, 125]. Similar concerns may also arise regarding the Bmf target regulation. Hence, a possible anoikis/apoptosis induction in healthy tissues and promotion of neurodegeneration or diabetes in response to drug-mediated Bmf upregulation should be taken in consideration in the future. However, Bmf may be considered as a predictive factor for assessing the effectiveness of cancer treatment, such as CLL. To conclude, “minor does not mean useless,” and this statement is true for the Bmf protein. Besides that, other functions of Bmf are likely to be unknown at this moment. Are they worth studying in the future? The answer is probably “yes” because, at least, it could shed light on the novel mechanisms underlying the physiological and pathological processes and, at most, improve existing therapeutic approaches. Altogether, research on distinct members of the Bcl-2 family proteins is of fundamental and applied significance, which is clearly shown for the Bmf protein here.
